# Underwater endoscopic submucosal dissection with pocket method and an additional working channel: from traction to closure

**DOI:** 10.1055/a-2783-4248

**Published:** 2026-02-17

**Authors:** Gabriele Altieri, Francesco Vito Mandarino, Jacopo Fanizza, Francesca Bernardi, Giuseppe Dell'Anna, Silvio Danese, Francesco Azzolini

**Affiliations:** 1 9372 Gastroenterology and Gastrointestinal Endoscopy Unit, IRCCS Ospedale San Raffaele, Milan, Italy; 2 476648 Gastroenterology and Gastrointestinal Endoscopy Unit, IRCCS San Donato Hospital, San Donato Milanese, Italy

Endoscopic submucosal dissection (ESD) is a safe and effective technique that allows en bloc resection of rectal neoplasia 1 . Several traction methods and dedicated devices have been developed to improve submucosal exposure, increase the dissection speed, and potentially reduce adverse events 2 3 4 .

**Fig. 1:**
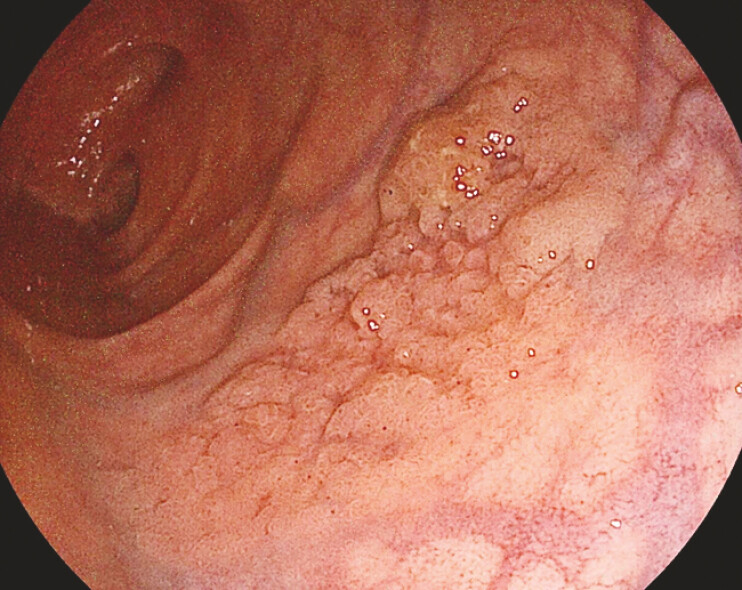
White light evaluation of the anterior rectal wall 25 mm LST-GH, with the adenomatous pattern and no signs of overt cancer.

**Fig. 2:**
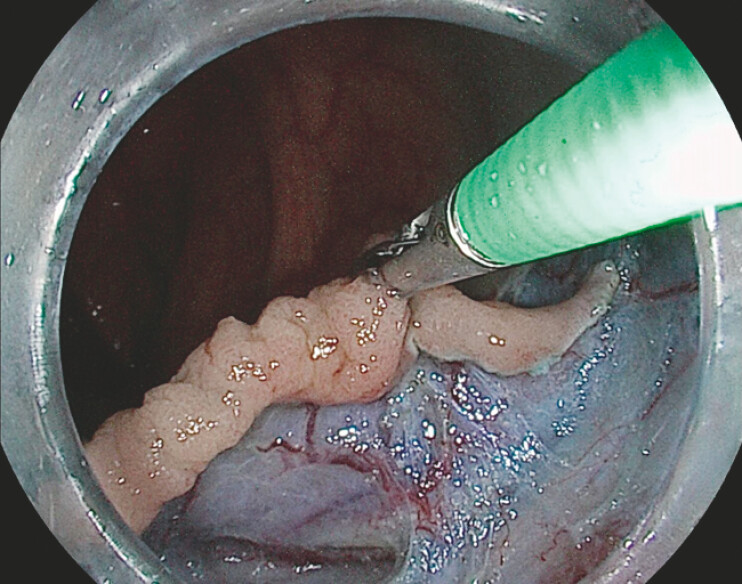
The implementation of an AWC enabled the usage of foreign body grasping forceps to apply traction, improving submucosal plane access and granting enhanced scope stability.

A 53-year-old man with no relevant medical history was referred for ESD of a 20 mm rectal laterally spreading tumor, granular homogeneous type, without endoscopic signs of invasive cancer ( Fig. 1 ). The procedure was performed in an underwater setting using the pocket technique as the primary dissection strategy, ensuring stable and controlled submucosal access ( Video 1 ). Within this framework, the AWC played a pivotal role by allowing the introduction of a foreign body grasping forceps into the submucosal tunnel, providing effective countertraction and improved visualization of the dissection plane, thereby facilitating a faster and more controlled dissection ( Fig. 2 ).

Underwater endoscopic submucosal dissection with the pocket method and an additional working channel to enable foreign body grasping forceps-assisted traction.Video 1

**Fig. 3:**
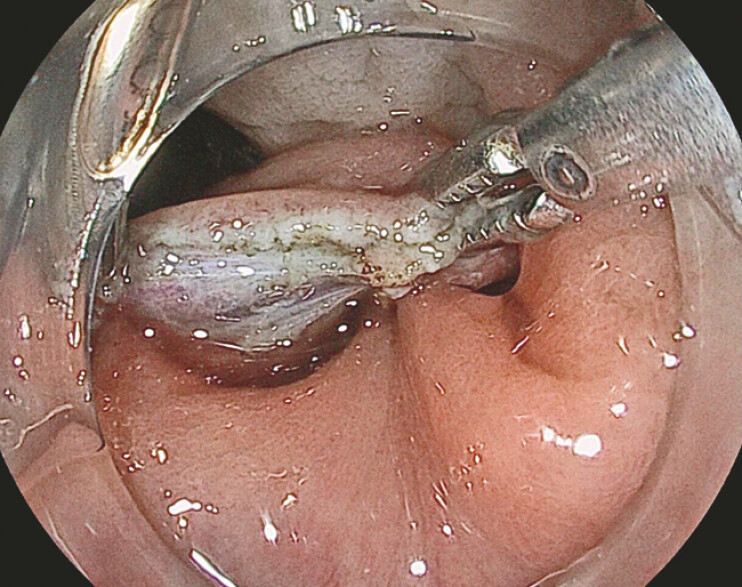
Post-ESD resection edges approximation facilitated by the through-the-AWC grasping forceps-assisted traction. ESD, endoscopic submucosal dissection.

After the completion of the resection, the same foreign body grasping forceps inserted through the AWC was used to approximate the resection margins, enabling the precise and efficient placement of through-the-scope clips for defect closure ( Fig. 3 ). The patient was discharged on the same day under good clinical conditions. Histological examination revealed a tubulovillous adenoma with low-grade dysplasia and R0 resection.

This case highlights how the AWC can enhance standard ESD techniques by supporting both effective traction within the submucosal tunnel and controlled closure of the resection defect. This simple and cost-efficient approach represents a practical alternative to more complex traction systems and emerging endoscopic robotic platforms.

Endoscopy_UCTN_Code_TTT_1AQ_2AC
